# N-Terminus-Mediated Solution Structure of Dimerization Domain of PRC1

**DOI:** 10.3390/cimb44040111

**Published:** 2022-04-10

**Authors:** Fei Tan, Jin Xu

**Affiliations:** School of Computer Science, Peking University, Beijing 100871, China

**Keywords:** protein regulator of cytokinesis, N-terminal domain of PRC1, solution structure, homodimerization, hydrophobic core packing, N-terminus-mediated core packing

## Abstract

**Simple Summary:**

The solution structure of the N-terminal domain of Protein Regulator of Cytokinesis 1 (PRC1) was determined, and compared with the previously published crystal structure, significant differences were found. Extensive analyses were carried out to find the true reason for the differences between the solution and crystal structures, we discovered that this might be related to the conformation of residue M1, which is buried in the protein core of the solution structure, while situated outside of the hydrophobic core in the crystal structure. In this study, we have carried out a series of examinations using various methods and confirmed that the N terminal conformation is the key point in better describing the structure of PRC1 dimerization domain under solution conditions.

**Abstract:**

Microtubule-associated proteins (MAPs) are essential for the accurate division of a cell into two daughter cells. These proteins target specific microtubules to be incorporated into the spindle midzone, which comprises a special array of microtubules that initiate cytokinesis during anaphase. A representative member of the MAPs is Protein Regulator of Cytokinesis 1 (PRC1), which self-multimerizes to cross-link microtubules, the malfunction of which might result in cancerous cells. The importance of PRC1 multimerization makes it a popular target for structural studies. The available crystal structure of PRC1 has low resolution (>3 Å) and accuracy, limiting a better understanding of the structure-related functions of PRC1. Therefore, we used NMR spectroscopy to better determine the structure of the dimerization domain of PRC1. The NMR structure shows that the PRC1 N terminus is crucial to the overall structure integrity, but the crystal structure bespeaks otherwise. We systematically addressed the role of the N terminus by generating a series of mutants in which N-terminal residues methionine (Met1) and arginine (Arg2) were either deleted, extended or substituted with other rationally selected amino acids. Each mutant was subsequently analyzed by NMR spectroscopy or fluorescence thermal shift assays for its structural or thermal stability; we found that N-terminal perturbations indeed affected the overall protein structure and that the solution structure better reflects the conformation of PRC1 under solution conditions. These results reveal that the structure of PRC1 is governed by its N terminus through hydrophobic interactions with other core residues, such hitherto unidentified N-terminal conformations might shed light on the structure–function relationships of PRC1 or other proteins. Therefore, our study is of major importance in terms of identifying a novel structural feature and can further the progress of protein folding and protein engineering.

## 1. Introduction

MAPs are a family of proteins that play crucial roles in cytokinesis in that they control the accurate division of cells into two daughter cells, collaborating with other factors to either stabilize or destabilize microtubules [1,2,3]. Protein Regulator of Cytokinesis 1 (PRC1) is an ideal representative of MAPs that stabilize microtubules by its role in motor-protein association and microtubule bundling [4,5]. PRC1 has two crucial functions, with the first one being microtubule binding and the second being self-association [6]. As PRC1 binds microtubules, motor proteins interact with PRC1 and drive it to the plus ends of microtubules [7,8,9]; PRC1 then pulls the microtubules together through self-association to build the spindle midzone, which is a complex system of MAPs and motor proteins loaded on microtubule filaments, to undergo cytokinesis [6,10]. Interferences with the function or the structure of PRC1 might abolish its function in segregating daughter cells and may result in the production of cancerous cells [7,11,12].

The full-length PRC1 protein has 620 residues, in which the 132 C-terminal residues are structureless and extremely susceptible to degradation, while its remaining 486 residues (also known as PRC1 1-486) possess the same function and cellular localization of the full-length protein [9]; therefore, PRC1 1-486 is generally used to represent full-length PRC1. The lack of structural data of most of the MAPs makes PRC1 an exceedingly intriguing protein to study, as the crystal structure of PRC1 1-486 has been published [13] (PDB ID 4L6Y). The crystal structure shows that the protein is an elongated rod-shaped homodimer, with each monomer in the homodimer consisting mainly of a coil-coil structure. The crystal structure can be divided into dimerization (aa: 1–65), rod (aa: 66–350) and spectrin domains (aa: 351–486). The spectrin domain comprises three helices, which form a spectrin fold. The rod domain is composed of six coil-coil helices with extensive contacts and interactions between the coils, this domain is also responsible for PRC1 microtubule binding through a conservative and positively charged stretch of residues. The dynamics of the rod domain were investigated in solution using molecular dynamic simulations, which showed that the rod domain of PRC1 exhibits modular flexibility in solution in order to bind antiparallel microtubules [13,14]. The dimerization domain (aa: 1–65) forms a U-shaped hairpin with two helices and one linker loop, upon dimerization, the two hairpins bisect and form a four-helix bundle, with extensive contacts between helices. Nevertheless, despite extensive characterizations of its structure, the self-multimerization properties of PRC1 remain mostly controversial and further studies in this domain are much needed [15].

The self-multimerization of PRC1 is essential for the microtubule-bundling activities of PRC1, various research groups have reported the multimerizational state of PRC1 and many were in conflict with each other. Zhu and Fu’s group found that PRC1 exists as a tetramer in unphosphorylated state, while Subramanian’s group stated that PRC1 is dimeric both in phosphorylated and unphosphorylated states [6,13,16]. Our group found that PRC1 exists mainly as homodimers. The above-mentioned 65 N-terminal residues of PRC1 are responsible for the multimerizational property of PRC1 and their deletion renders PRC1 a monomer.

For most proteins, the N terminus is often exposed and thrown out of the hydrophobic core, as it is insignificant to the overall packaging of the protein. However, for some proteins, such as SARS-CoV Main protease [17,18], Xylanase (BSX) from *Bacillus* sp. [19,20], Flock house virus (FHV) coat protein [21], etc. [22,23], it has been suggested that the N-terminal regions play important roles in the function or the folding of the molecule. Seven N-terminal residues of SARS-CoV Main protease form an N finger, which is crucial for maintaining the protein’s enzymatic activity in the cleavage of viral coat proteins, while additional residues to its N terminus disrupt its ability to form enzymatically active dimers. BSX is resistant to degradation by both SDS and proteinase K; extensive analyses revealed that this poly-extremophilicity was provided by hydrophobic interactions mediated by its N terminus, mutations in N-terminal residues abolish its resistance to SDS and proteinase K degradation. FHV coat proteins participate in the assembly of FHV, the N terminus of FHV coat proteins contain determinants for the recognition and packaging of viral RNA, and the deletion of N-terminal residues 2–30 resulted in a chaotic viral-RNA production pattern, producing malformed viruses. In this study, we observed a special conformation in the N terminus of PRC1, which plays essential roles both in the structure and in the folding of the whole protein.

The precise characterization of protein solution structures is integral for understanding the relationship between protein structure and function under solution conditions [24]. The low resolution (the data collection of the crystal structure PRC1-1-486 was made to 3.2 Å) and the incomplete sidechains of the PRC1 crystal structure, together with its high B-factors in part of the structure, motivated us to probe the precise solution structure of the PRC1 dimerization domain using nuclear magnetic resonance (NMR) spectroscopy [15].

In this study, we separately expressed the PRC1 dimerization domain (also known as PRC1-DD; aa: 1–65), which is an independent protein domain with few or no interactions with distal parts of PRC1. PRC1-DD is a stable homodimer with molecular mass of around 15 kDa which remains stable even under 8M urea (Supplementary Information (SI), Figures S1–S3). Here, we report the three-dimensional solution structure of PRC1-DD determined by NMR spectroscopy and the differences between the solution structure and corresponding segment in the crystal structure. We thoroughly analyzed the differences between the solution and crystal structures. One of the most distinguishing features of PRC1-DD is its N terminus, in which the first residue is folded into the protein core, participating in hydrophobic interactions with other core residues. We confirmed this structural trait with mutational studies [25,26,27,28,29,30] and fluorescence thermal shift assays, which measured the structure and stability of the protein. Based on these results, we proved the superiority of the solution structure in describing the solution-state conformation with respect to the crystal structure.

## 2. Materials and Methods

### 2.1. Protein-Template Construction

The nucleic acid sequences of PRC1 1-486 and PRC1-DD were amplified from human-PRC1-isoform-1 (Pubmed accession: NP_003972) and inserted into the bacterial expression vector pET-21a containing a C-terminal His-tag. The detailed protocol is as follows:

1. DNA fragments encoding PRC1 1-486 and PRC1-DD were amplified by polymerase chain reaction (PCR) with primer sequences shown in Table S3.
2. The PCR products and the circular pET-21a vector were digested by restriction enzymes Nde I and Xho I (NEB), respectively, to obtain target gene insertion fragments with sticky ends and the corresponding linear plasmids.
3. The insertion fragments of the target gene were incorporated into the linear plasmid vector by T4 ligase and the product was transferred to *E. coli* competent cell TOP10. The positive strain was screened by ampicillin resistance, and PCR confirmation and DNA fragment sequencing were carried out.
4. The recombinant plasmids with correct sequencing were obtained using a plasmid extraction kit (Invitrogen).

### 2.2. Mutant-Plasmid Generation

1. Mutant proteins of PRC1 1-486 and PRC1-DD were generated using a QuickChange Mutagenesis kit (Agilent) with the following reaction system: 200 ng of forward primer, 200 ng of reverse primer (provided in Table S3), 1 μL of 5 *reaction buffer, 10 μL of 2. 5 mM dNTPs, 4 μL of Pfu DNA polymerase and 1 μL of Nuclease-free water, for a total of 50 μL.
2. We set the PCR reaction conditions according to the instructions of the Pfu DNA polymerase for 30 cycles.
3. We added DPN1 restriction endonuclease to the reaction system and incubated it at 37° for 1 h.
4. We tested the products by electrophoresis and selected the DNA band with the correct length and used a DNA gel Recovery Kit (Zymo Research) to obtain the target plasmid; then, we used 100 ng of the product for transformation into *E. coli* competent cell TOP10. Antibiotic agar plates for positive-strain selection were used.
5. Single colonies were picked and sent for sequencing. The correct plasmids were used for mutant protein expression.

### 2.3. Protein Expression

Expression of wild-type PRC1-DD, PRC1 1-486 and mutant proteins were carried out in *E. coli* BL21 (DE3) or *Rosseta* (DE3) cells. Proteins were expressed for 6–20 h at 18–35 ${}^{{}^{\circ}}$C with 500 mM IPTG and then purified in accordance with the following protocol:

1. The recombinant plasmids encoding the PRC1 target proteins were transformed into *E. coli* competent cells (both BL21 (DE3) and *Rosseta*); the plates were coated with agar containing ampicillin and incubated at 37 ${}^{{}^{\circ}}$C.
2. Single colonies were selected and inoculated into 40 mL of Luria Bertani (LB) medium containing ampicillin, then incubated overnight at 35 ${}^{{}^{\circ}}$C.
3. We transferred the bacterial solution into 1 L of fresh LB medium containing ampicillin and cultured it at 35 ${}^{{}^{\circ}}$C to OD 600; then, we added 0.05 g of IPTG to induce the expression of the target protein at 18 ${}^{{}^{\circ}}$C for 6–20 h.
4. After continuous culturing for 6 h, we centrifuged the bacterial solution at 7000 rpm for 15 min, poured out the supernatant and resuspended the bacteria with 30 mL of liquid buffer containing 50 mM Sodium Phosphate monobasic/dibasic and 300 mM NaCl, pH 8.0; then, we froze it at −80 ${}^{{}^{\circ}}$C.

### 2.4. Protein Purification

1. *E. coli* cells were harvested by centrifugation, resuspended in lysis buffer (50 mM Sodium Phosphate, 300 mM NaCl, 5% glycerol and 25 mM imidazole, pH 8.0), supplemented with protease inhibitor cocktail (Roche, Indianapolis, IN, USA) and then sonicated.
2. After separation of supernatant and pellet by centrifugation, the supernatant was loaded onto a His-Trap HP column (Tiagen, Beijing, China). We pooled the His-tagged proteins by applying a linear imidazole gradient (20–300 mM).
3. The proteins were then further purified by size-exclusion chromatography on a Superdex 75 or Superdex 200 column (GE Healthcare Life Sciences, Piscataway, NJ, USA) equilibrated at 50 mM Sodium phosphate and 150 mM NaCl, pH 7.0. Fractions corresponding to the target proteins, as confirmed by SDS-PAGE, were pooled, concentrated and stored at −80 ${}^{{}^{\circ}}$C. The purity of all protein preparations was greater than 95% based on polyacrylamide gel electrophoresis in the presence of DTT.

### 2.5. Labeled-Protein Preparation

We used *E. coli* BL21 (DE3) or *Rosseta* cells for labeled-protein expression. To prepare ${}^{15}N$-labeled or ${}^{15}N$/${}^{13}C$-labeled protein for NMR studies, we grew cells in M9 minimal medium with ampicillin (100 mg/L) and ${}^{15}NH_{4}Cl$ in the absence or presence of ${}^{13}C$-glucose for the generation of ${}^{15}N$-labeled or ${}^{15}N$-${}^{13}C$-double-labeled samples [31].

After overnight incubation at 37 ${}^{{}^{\circ}}$C in LB growth medium, the cells were added in a 1:20 ratio by volume to 1 L of LB medium. After cells reached an absorbance of 0.8–1.0 at 600 nm (around 4 h at 37 ${}^{{}^{\circ}}$C), they were added to 500 mL of M9 minimal medium with ${}^{15}NH_{4}Cl$ and D-glucose-1,2,3,4,5,6,6-d7 as the sole nitrogen or carbon sources. All media contained 100 mg/L ampicillin. After the cells had grown at 37 ${}^{{}^{\circ}}$C to an absorbance of 0.8 at 600 nm, the temperature was decreased to 18 ${}^{{}^{\circ}}$C and IPTG was added at a concentration of 100 mg/L. After 6–20 h of protein expression, cells were harvested by centrifugation. The PRC1 protein was then extracted from cells and purified.

### 2.6. High-Performance Chromatography

A high-pressure liquid chromatography system was equipped with a UV detector (Hitachi, Tokyo, Japan). The HPLC columns were Superdex 75 5/150 and Superdex 200 5/150 (GE Healthcare Life Sciences).

The protein sample (<10 mg) was dissolved in effluent and injected into the column with a flow rate of 0.3–1 mL, as recommended by the column manual, and before injection, all samples were centrifuged at 10,000× *g* for 10 min. All figures and molecular mass measurements were generated directly by the device’s default software.

### 2.7. Chemical Cross-Linking

Cross-linking studies were carried out using PEG5 as cross-linker, which cross-links every possible NH ester within 23 Å. Prior to cross-linking, the purified proteins were kept in buffer containing 50 mM sodium phosphate, 150 mM NaCl, pH 7.4. Sample concentration was adjusted to 20 nM and 50 nM, then cross-linking reactions were carried out at 25 ${}^{{}^{\circ}}$C with 0.1 M–1 M PEG5 reagent (Thermo Fisher Scientific, Waltham, MA, USA). Aliquots were removed after 30 min of incubation and reaction-quenched by the addition of 1M Tris-HCl, to a final concentration of 50 mM. The reaction results were tested by SDS-PAGE [32].

### 2.8. NMR Sample Preparation

NMR samples were prepared in 50 mM Sodium Phosphate, 150 mM NaCl, 5 mM DTT, 0.01% DSS and 5% $D_{2}O$, pH 7.4, and were between 0.5 mM and 1.0 mM in concentration. Isotopic heterodimers were prepared by mixing equal amounts of unlabeled and ${}^{15}N$-${}^{13}C$-double-labeled protein (16 mg each) in 2 mL of 8 M urea for several minutes and then dialyzing by ultracentrifugation (Amicon Ultra Centrifugal Filters; 10,000 molecular weight cutoff). The samples were then concentrated to 1 mM. All steps were performed at 4 ${}^{{}^{\circ}}$C.

### 2.9. Resonance Assignments

NMR experiments were carried out at 298 K on Bruker Avance 500 and 800 MHz spectrometers using cryogenically cooled probes equipped with ${}^{13}C$- and ${}^{15}N$-decoupling and pulsed-field gradient coils. All NMR spectra were processed using NMRPipe [33] and analyzed using NMRView [34]. Two-dimensional ${}^{1}H{-}^{15}N$ HSQC and triple-resonance experiments, including three-dimensional HNCAC [35], CBCA(CO)N [36], HNCA and HN(CO)CA [37], were performed to carry out backbone chemical shift assignments of PRC1-DD. Two-dimensional ${}^{1}H{-}^{15}N$ HSQC, three-dimensional (H)CC(CO)NH [38] and (H)CCH-TOCSY [39], (H)CCH-COSY [40], HCCH-TOCSY [39] and HCCH-COSY [41] experiments were performed to obtain the backbone and side-chain chemical shift assignments of PRC1-DD. Three-dimensional ${}^{15}N$- and ${}^{13}C$-edited NOE-HSQC spectra (mixing time, 150 ms) were collected to confirm the chemical shift assignments and generate distance restraints for structure calculations [42,43,44].

### 2.10. Structure Calculations

The analyses of 3D ${}^{15}N$- and ${}^{13}C$-edited NOESY experiments were carried out for the identification of intramolecular NOE distance restraints. Isotopic heterodimers were formed from mixing equal amounts of unlabeled and ${}^{15}N$–${}^{13}C$-labeled PRC1-DD; ${}^{15}N$–${}^{13}C$-edited NOESY experiments, with a mixing time of 150 ms, were performed to identify strictly intermolecular NOE distance restraints.

The structure calculations were initially performed with the torsion-angle dynamic algorithm DYANA (CANDID structure calculation suite [45]) as a first calculation step and further refinements were carried out using the program AMBER [46]. TALOS [47] was used for obtaining backbone Φ and Ψ restraints by analyzing backbone chemical shifts. All distance restraints were generated using the program SANE [48]. The final 20 structures with the lowest AMBER energy were selected for Protein DataBase deposition (PDB ID 7VBG). PROCHECK_NMR [49,50] was used for analyzing the quality of the structure.

### 2.11. Fluorescence-Based Thermal Shift Assay (FTSA)

Thermal shift assays were performed using a Real-Time PCR Detection System (StepOne Real-Time PCR System, Applied Biotechnology, San Luis Obispo, CA, USA) with a temperature increment of 0.2 ${}^{{}^{\circ}}$C and a temperature range of 25–95 ${}^{{}^{\circ}}$C. A total of 25 μL of mixtures containing 2.5 μL of protein dye (Protein Thermal Shift Starter Kit, Life Technologies, Carlsbad, CA, USA; diluted from 5000× g concentrate stock), 10 μL of reaction buffer (Protein Thermal Shift Starter Kit) and 12.5 μL of protein (at a concentration of 0.5 mM), was mixed on ice in a 96-well plate. The mid-denaturation temperatures (Tm) that measure protein folding and unfolding transitions were estimated using the device with the following equation [51,52,53]:

(1) $$I=(A+\frac{B-A}{1+e(T_{m}-T)/C})$$

where *I* is the fluorescence intensity at temperature *T*, *A* and *B* are the pre-transitional and post-transitional fluorescence intensities, respectively, and *C* is a slope factor [54].

### 2.12. Static Light Scattering and SEC-MALS

First, we prepared the target protein samples in a gradient of concentration from 0.1 to 1 mM; second, we filtered the samples through a membrane with a pore diameter of 0.1 μm and degassed it with argon. Finally, the protein samples were loaded onto the chromatography columns and eluted at 0.5 mL/min. The absorbance and/or refractive indices were measured and the accurate quantification of macromolecular mass was carried out using the Debye method on the ALV CGS-3 dynamic/static-light-scattering device.

### 2.13. Statistical Analysis

The statistical analyses were carried out using the paired two-sample Student’s *t*-test for means in Excel, comparing each mutant with the wild type. The *p*-value thresholds were set at 0.05 for significant and at 0.01 for very significant.

## 3. Results

**Figure 1:**
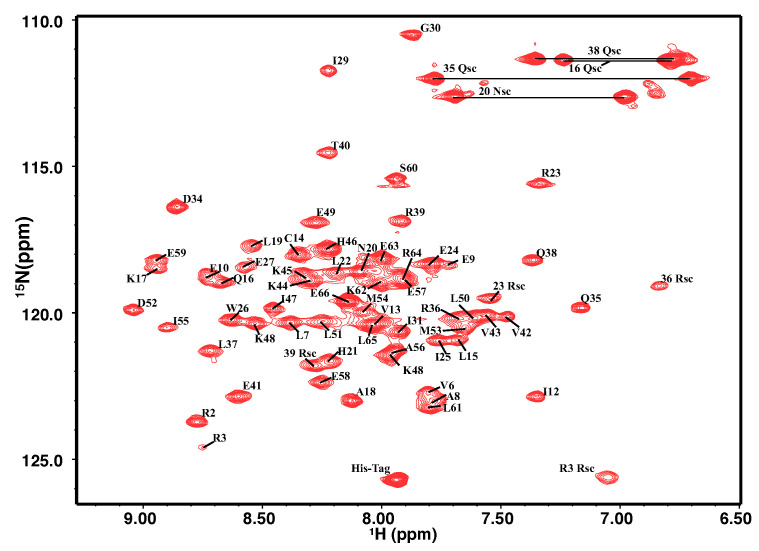
Two-dimensional ${}^{1}H{-}^{15}N$ HSQC spectra and assignment of PRC1-DD.

The construct PRC1-DD, which we used for structural determination, was an independent domain in full-length PRC1. Through chemical cross-linking, size-exclusion chromatography and static-light-scattering analysis and pulsed-field diffusional studies (Figures S1 and S2), we identified PRC1-DD as a thermally and structurally stable (Figure S3) homodimer in solution with 65 residues per subunit (Figure 1).

### 3.1. Three-Dimensional Structure of PRC1-DD

The 2D ${}^{1}H{-}^{15}N$ HSQC spectra of the homodimeric PRC1-DD are displayed in Figure 1. The NH signals of 61 out of 64 non-proline residues were assigned (except S4, S11 and E33); the missing residues were possibly caused by exchange broadening [55,56]. The secondary structure of PRC1-DD was composed of two α-helices, one loop. The largely α-helical nature of this protein could be predicted both in the characteristic pattern of cross peaks in 3D ${}^{1}H{-}^{13}C$ NOESY spectra and in the backbone dihedral angles speculated from chemical shifts [57]. The inter-helical packing arrangement was unambiguously determined by unique inter-monomer distance constraints. The solution structure of PRC1-DD was calculated based on 3717 intra-subunit distance restraints together with 120 inter-subunit distance restraints.

**Figure 2:**
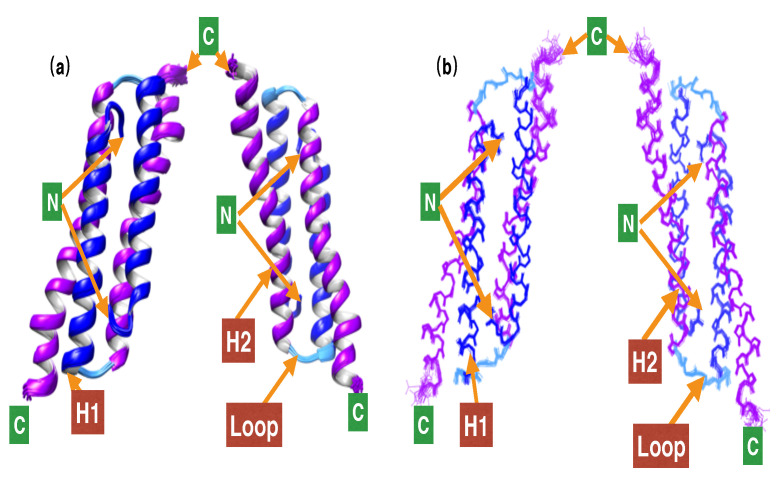
Solution structures of PRC1-DD. (**a**) Ribbon representation of the 20 lowest-energy structures. (**b**) Backbone chain trace of the 20 lowest-energy structures; helices H1 and H2 from the two monomeric units in the homodimer are colored separately in blue (H1, M1-E28), cyan (loop, I29-D32) and purple (H2, E34-L65); C and N tags signify C- and N-terminal ends, respectively. This and all other structural figures were generated using the program CHIMERA.

The restraint and structural statistics for the ensemble of the 20 most energetically favorable structures are given in Table 1. The solution structure of PRC1-DD is well-defined, with an average root-mean-square deviation (RMSD) of 0.49 Å for all the backbone heavy atoms. A ribbon diagram of the 20 lowest-energy structures is shown in Figure 2.

Each subunit of PRC1-DD contains 65 residues that form a U-shaped hairpin with two α-helices (H1 and H2) formed by residues R3-E27 and R35-K62, respectively, and one loop (L) formed by L28-Q34. Upon dimerization, hairpins bisect each other to form a four-helix bundle. A dimeric interface is created by H1s and H2s of two different protomers. Superimposing one monomeric unit with another resulted in an RMSD of 0.43 Å for the backbone heavy atoms, indicating a well-defined dimeric interface.

### 3.2. Comparison between Solution Structure and Crystal Structure

**Figure 3:**
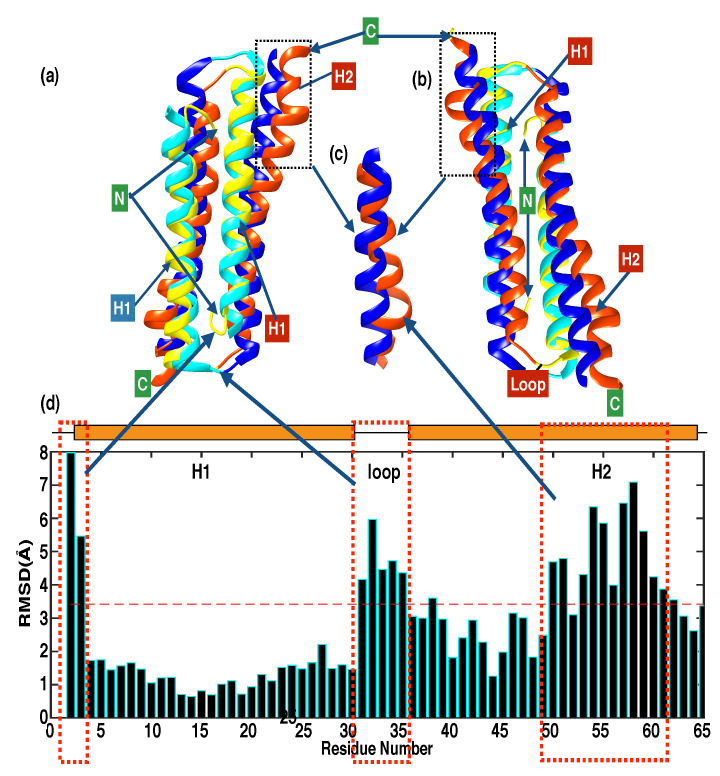
Comparison of the solution structure of PRC1-DD and the corresponding segments in the crystal structure. (**a**) Superimposition of ribbon representation of the PRC1-DD lowest-energy solution structure and crystal structure. H1 and H2 of the solution structure are colored yellow and orange, respectively, while H1 and H2 of the crystal structure are colored cyan and blue, respectively. C and N tags signify C- and N-terminal ends, respectively. (**b**) A different orientation of (**a**). (**c**) Enlargement of the dotted box in (**a**,**b**). (**d**) Bar graph showing the RMSD values of residues between solution structure and the corresponding segment in the crystal structure for a single monomeric unit when aligning all backbone heavy atoms. Figures were plotted using MATLAB; the alignments were performed using CHIMERA.

The solution structure of PRC1-DD and the corresponding segment in the crystal structure of PRC1 1-486 have the same helix-turn-helix motif, but significant differences are still evident. In terms of secondary structure, H1 starts from E5 in the solution structure, compared with R3 in the crystal structure; moreover, H2 starts from D34 in the solution structure, while it starts from E33 in the crystal structure. When aligning the solution and crystal structures, the overall RMSD for backbone heavy atoms resulted to be 3.48 Å in which the RMSD over residues 1–28 (H1) was calculated to be 1.67 Å, indicating relatively good similarity in terms of helix H1, while the RMSD over residues 34–62 of helix H2 was found to be 3.67 Å. Significant structural differences between the solution and crystal structures are shown in the superposition structure in Figure 3a. The largest distinctions are in the N terminus, the loop area (residues E27–P32) and residues E49–E58 (Figure 3d). N-terminal M1 is situated inside the hydrophobic core and is a unique trait of the solution structure, with an RMSD value of 7.51 Å compared with M1 of the crystal structure. The loop area gave a high RMSD value, averaged around 4.01 Å; residues E49–E58 of H2 differ from the crystal structure with an average RMSD value of 4.75 Å (Figure 3c). The fact that there are areas that exhibited large RMSD values between the solution and crystal structures suggests that, in PRC1-DD, these areas may adopt different conformations under solution and crystalline conditions.

Aligning helices H1 of the solution and crystal structures resulted in an even lower RMSD for H1 (1.07 Å) and slightly higher RMSD values of 4.58 Å and 4.06 Å for the loop area and H2, respectively (Figure S4a). This method of comparison in addition to the first confirmed that the loop area and H2 of PRC1-DD exhibit the largest differences between the solution and crystal structures. Furthermore, even the dimerization interfaces of the solution and crystal structures are different, fitting a single monomeric unit in the homodimer, which produced an average RMSD of 2.61 Å for one monomeric unit, while generating a large RMSD of 5.72 Å for the other, thus suggesting that the two structures even possess distinctive dimerizational interfaces (Figure S4b).

**Figure 4:**
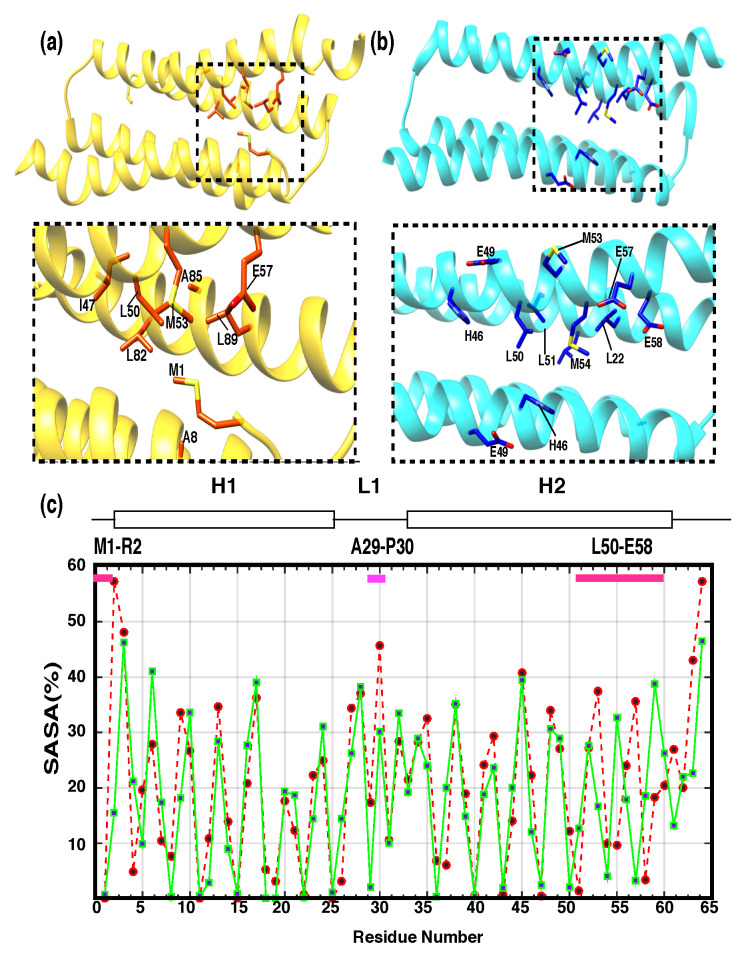
Differences between solution and crystal structures. (**a**,**b**) The hydrophobic core composition of (**a**) solution and (**b**) crystal structures with residues having significant differences being tagged. (**c**) Relative solvent-accessible surface area (SASA) as a function of residue number (*x*-axis); each residue’s SASA value is represented as a black dot, the green continuous line belongs to the SASA values of the crystal structure, while the dotted red line represents solution-structure SASA values.

Even though the overall assembly of the solution and crystal structures of PRC1-DD are similar, their hydrophobic core packing differ significantly. Residues that differ the most between the two structures are mostly centered around the N terminus, the loop area and the end of helix H2; these differences contribute to the large differences in the core packing of the structures. As indicated in Figure 4, when comparing the crystal and solution structures, residues such as M1, R2, L50, L51, M53, M54, E57, E58 and E59 differ vastly in terms of their position with respect to the hydrophobic core; in fact, M1, R2, L50, M53 and E57 are hydrophobic core residues according to the solution structure, while these residues are located outside the hydrophobic core in the crystal structure. In contrast, residues L51, M54, E58 and E59 are situated inside the hydrophobic core according to the crystal structure, while they are more solvent exposed as indicated by the solution structure. These controversies demonstrate that the residues participating in hydrophobic core formation are significantly different in the crystal and solution structures. Accordingly, the relative solvent-accessible surface area (SASA) calculated for both structures (Table S1) also confirmed the distinctive hydrophobic core composition of the two structures, with different hydrophobicity patterns among the above-mentioned residues (Figure 4c).

In addition, the 3D ${}^{1}H{-}^{13}C$-NOESY cross peaks and residue orientations generated by the Residual Dipolar Coupling (RDC) data confirmed the different orientations exhibited by residues M1, L50, L51, M53, M54, E57 and E58 in the solution and crystal structures. One of the reasons why the calculated solution structure ensemble is different from the crystal structure might originate from the fact that some characteristic distance restraints and orientation restraints distinguish the solution structure from the crystal structure [58]. As shown in Figure S5 and Table S2, the cross peaks in the 3D ${}^{1}H{-}^{13}C$-NOESY spectra signify that the distance between the two protons in the solution structure is within 6 Å, while on the other hand, the distance between the corresponding protons in the crystal structure is far greater than 6 Å; therefore, the mere existence of these 3D ${}^{1}H{-}^{13}C$-NOESY cross peaks indicates that the core packing of the solution and crystal structures is significantly different.

### 3.3. The N-Terminal Extension of PRC1 Crystal Structure

**Figure 5:**
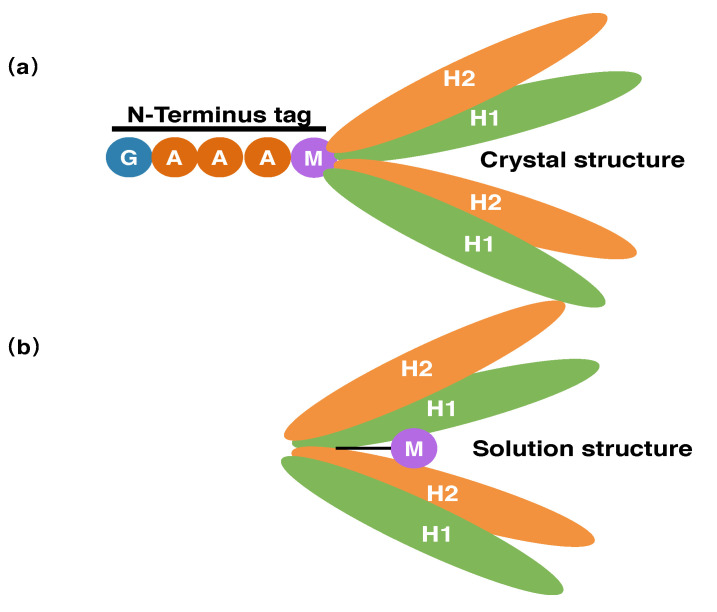
Schematic diagram of PRC1-DD (**a**): crystal and (**b**) solution structure.

**Figure 6:**
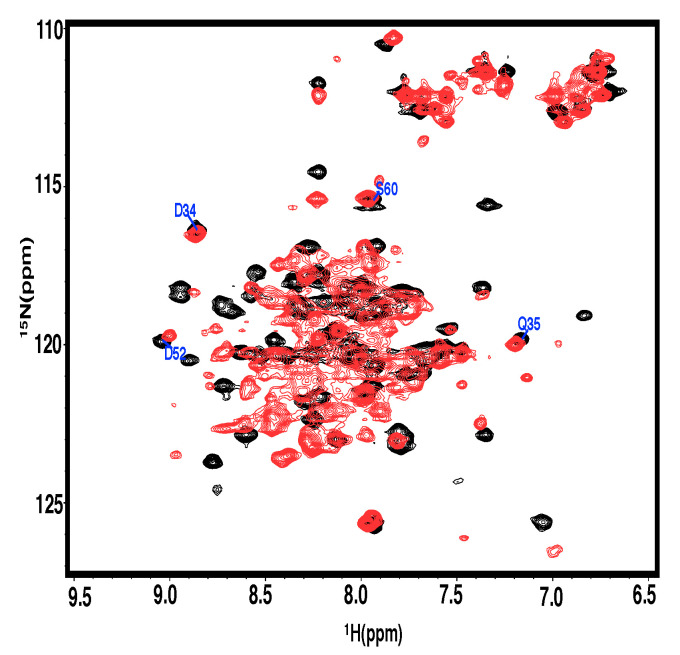
Two-dimensional ${}^{1}H{-}^{15}N$ HSQC spectra of N-terminal extension mutant (red) and wild type (black).

In the solution structure of PRC1-DD, the most prominent feature is residue M1, which is stretched into the protein core, making extensive contacts with the hydrophobic residues in the core, possibly playing a crucial role in maintaining the stability of the whole protein. However, when we examined the PRC1 construct used for crystal-structure determination, we found that an additional sequence of ‘GAAA’ was attached to the N terminus of PRC1, and it is very likely that this extra N-terminal tag renders residue M1 unable to stretch into the hydrophobic core due to spatial constraints, affecting the correct core packing of the structure and causing the different conformations of the solution and crystal structures (Figure 5). In order to determine if the extra N-terminal tag indeed affected the overall protein structure, we created N-terminal extension mutants PRC1-DD-N+4 and PRC1-DD-N+His by engineering a sequence of ‘GAAA’ and His-tag, respectively, into the N terminus of wild-type PRC1-DD. As a result, the expression levels of the extension mutants were significantly lower than those of the wild type (Figure S7); moreover, the 2D ${}^{1}H{-}^{15}N$ HSQC spectra of those N-terminal-modified mutants also showed huge alterations, with almost all cross peaks being moved from their original position (only the chemical shift of 3–4 residues remained unchanged). The NMR spectra also exhibited attributes of protein aggregation, with a large number of overlapping cross peaks in the center of the spectra (similar to Figure 6), indicating that the N-terminal extension not only caused huge structural changes in PRC1-DD, but also affected its correct folding.

### 3.4. Structural and Thermodynamical Significance of N Terminus of PRC1-DD

Since all the differences between the solution structure and the crystal structure were possibly the result of the special conformation adopted by the N-terminal residues, we employed mutational studies and fluorescence thermal shift assays to reveal the exact conformation of the PRC1 N terminus under solution conditions.

**Figure 7:**
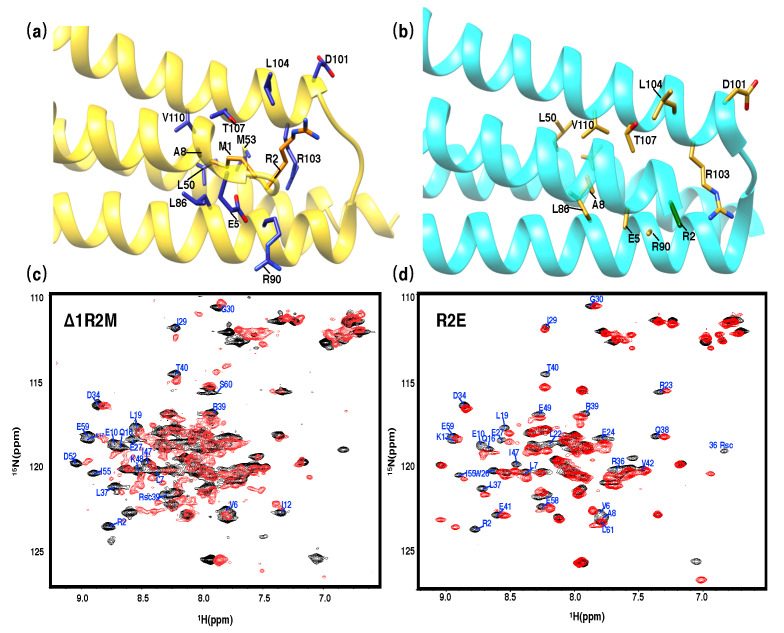
Two-dimensional ${}^{1}H{-}^{15}N$ HSQC spectra of Δ1R2M and R2E. Ribbon representation of the (**a**) solution structure (gold) and (**b**) crystal structure (cyan) with the side chains M1 and R2 in orange and green, while the side chains of the residues interacting with M1 or R2 are shown in blue and mud yellow for solution and crystal structures, respectively. (**c**) Two-dimensional ${}^{1}H{-}^{15}N$ HSQC spectra of Δ1R2M. (**d**) Two-dimensional ${}^{1}H{-}^{15}N$ HSQC spectra of R2E; residues experiencing great chemical shift changes (Δ comp ≥ 0.3 ppm) are labeled.

Residue M1 is stretched into the protein hydrophobic core in the solution structure, while it is situated entirely outside the protein core according to the crystal structure. The conformation of M1 in the solution structure is supported by many short- and medium-range (4–5 Å) NOESY cross peaks (Figure S5, Table S2) observed between residue M1 and core residues L50 and M53 of the same monomer and residue V43 and L19 of the other monomeric subunit. We created a series of mutants to assess the role that M1 plays in stabilizing the hydrophobic core, mutant 1ΔR2M deleted residue M1 and changed residue R2 to methionine. The 2D ${}^{1}H-$${}^{15}N$ HSQC spectra of 1ΔR2M experienced significant changes compared with the wild type, whereby most peaks in the spectra were moved far from their original positions (Figure 7); for instance, residues in the spatial vicinity of M1 according to solution structure, such as R3, A8, L19, T40, R36, L22, L37, R39, V47, M53 and M54, all exhibited significant chemical shift perturbations (complex chemical shift changes (Δcomp ≥ 0.3 ppm)) [55]; thus, we can conclude that PRC1-DD experienced substantial structural variation in the core area when we deleted the first residue. We speculate that, if the first residue were to be placed outside the protein core according to crystal structure instead, deleting M1 would not have had such a large impact on the overall structure of the protein.

To exclude the possibility that mutant 1ΔR2M changes the structure of PRC1-DD by affecting the residue composition of R2, we generated mutant R2E by mutating R2 to a negative-charged residue E and keeping M1 unchanged. Mutating R2 affected the solution structure by changing the electrostatic environment of the protein core, which was reflected in the 2D ${}^{1}H{-}^{15}N$ HSQC spectra of R2E, in which residues that according to the solution structure are located close to R2, such as W26, E27, R36, R37 and T40, experienced great chemical shift changes. However, W26, E27 and T40 were further away from R2 in the crystal structure, and such structural alterations could not be supported by the crystal structure. Generally speaking, the spectral differences between R2E and wild type were significantly smaller than those between 1ΔR2M and wild type, signifying that the large structural change observed in 1ΔR2M was the result of the deletion of M1 rather than the effect of subsidiary changes made in R2. We labeled the residues with substantial chemical shift changes in Figure 7 and mapped the corresponding residues to the structure in Figure S6. It is evident that only the residues around R2 displayed higher chemical shift changes. Therefore, even though the mutation in R2 caused certain changes in the PRC1-DD structure, these changes were compatible with the solution structure and the significance of M1 to overall structural integrity is indisputable.

**Figure 8:**
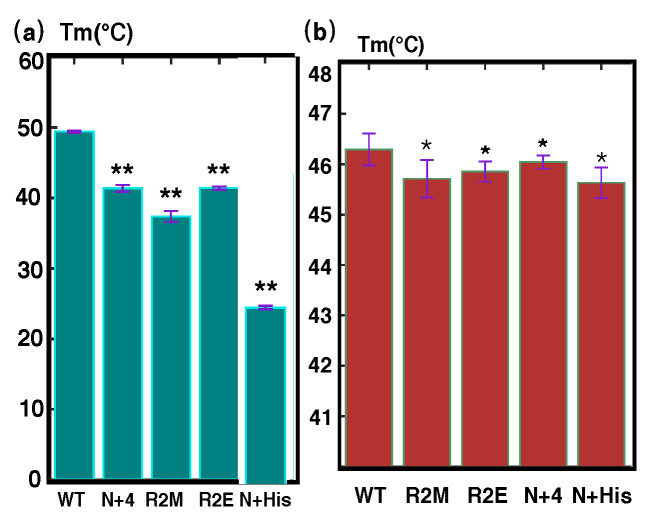
Fluorescence thermal shift assay of PRC1-DD, PRC1-486 and its mutants (bar graph representation of data in Table 2 and Table 3). (**a**) Bar graph showing the mid-denaturation temperature (Tm) of wild-type PRC1-DD and its mutants. The name of each mutant is labeled on the *x*-axis of the figure. (**b**) Bar graph showing the mid-denaturation temperature (Tm) of wild-type PRC1 1-486 and its mutants. The name of each mutant is labeled on the *x*-axis of the figure; R2M refers to Δ1R2M. Graphs were produced in MATLAB, plotted from the mean and standard deviations (thin red T) of Tm from four parallel experiments; single star indicates statistical significance (p<0.05) and double star indicates strong significance (p<0.01).

In addition to using NMR studies to assess the structural changes brought by mutating N-terminal residues, we also measured the thermodynamic stability of the PRC-DD and its N-terminal mutants by fluorescence-based thermal shift assays (FTSAs) (Table 2 and Figure 8). The FTSA measures the mid-denaturation temperatures (Tm) of proteins, which is a standard metric quantifying the stability of a protein. We observed relatively large decreases in the thermal stability of PRC1-DD N-terminal mutants compared with the wild type (Figure 8b). The Tm of mutant 1ΔR2M was 10 ${}^{{}^{\circ}}$C lower than that of the wild type, while N-terminal extension mutants PRC1-DD-N+4 and PRC1-DD-N+His also possessed significantly lower Tm values (more than 8 ${}^{{}^{\circ}}$C lower than the wild type), indicating that the stability of PRC1-DD was substantially reduced by N-terminal modifications. In sum, the adversely affected thermal stability of M1 mutants, together with the fact that mutant R2E was much more stable than 1ΔR2M, further supports the essential role that M1 plays in stabilizing the PRC1-DD protein hydrophobic core.

### 3.5. Assessing N-Terminal Conformation of Full-Length PRC1

Even though PRC1-DD is an independent protein domain in terms of structure and function, it is nonetheless a truncated segment of the full-length protein; therefore, the question of whether the full-length protein possesses the same structural properties of PRC1-DD still remained. To address this problem, we engineered N-terminal mutations R2M, R2E, N+4 and N-His into PRC1 1-486 (aa: 1–486), which represents full-length PRC1.

The thermal stability of wild-type PRC1 1-486 and its mutants were measured by FTSAs (Figure 8 and Table 3). Agreement in the pattern of thermal stability could be observed between the mutants of PRC1 1-486 and PRC1-DD. Consistent with the solution structure, N-terminal mutants PRC1 1-486ΔR2M, PRC1 1-486-R2E, PRC1 1-486-N+4 and PRC1 1-486-N+His affected the stability of PRC1 1-486 in the same way as the corresponding mutations in PRC1-DD, even though to a lesser extent. In sum, all of the above mutational studies demonstrated that PRC1-DD and full-length PRC1 showed a strong possibility of possessing the same N-terminal structural traits, with residue M1 being placed inside the hydrophobic core and contributing to core stability, thus excluding the likelihood that the N-terminal structural disconformities between the solution and crystal structures were a consequence of protein truncation.

## 4. Discussion

PRC1-DD is responsible for the dimerization of PRC1. It is an independent domain, with few or no interactions with the distal parts of the full-length protein according to the crystal structure; therefore, we separately expressed PRC1-DD and determined its solution structure. The solution structure of PRC1-DD has high resolution (in overall backbone RMSD) for the backbone heavy atoms (0.37 Å) compared with the crystal structure (3.1 Å).

By comparing the solution and crystal structures of PRC1-DD, we found that they differed significantly in terms of folding and hydrophobic core compositions. The mutational studies on N-terminal residues revealed that the solution structure better reflected the conformation that PRC1 adopted under solution conditions. Here, we discuss the implications of the results shown in the last section and presented the possible reasons for the differences between the two structures.

Firstly, the analyses of the PRC1 crystal structure showed that the N terminus and helix H2 exhibited greater-than-average crystal B-factors (Figure S8). The B-factor is a measure of structural uncertainty induced by protein dynamics and thermal disorder [59,60], greater-than-average B-factors indicate that the PRC1 N terminus and H2 might possess greater-than-average protein flexibility and conformational disorders. This greater flexibility might facilitate conformational rearrangement, causing the different conformations of PRC1 under solution and crystal conditions [61]. In addition, a previously reported cryo-electron microscopy analysis showed that PRC1-DD lacked electron density [13,14], suggesting that PRC1-DD is an inherently dynamic structure and may assume different conformations under different environmental conditions.

Secondly, the N terminal of the protein possesses a unique structural trait, with residue M1 inserting into the hydrophobic core. Generally, protein N-terminal residues should be flexible and stretched out of the hydrophobic core, being insignificant to the overall hydrophobic packing of the protein; however, for other proteins, such as SARS-CoV Main protease (Mpro), BFX, FHV coat protein, etc., the first residue is indispensable to protein function or structural integrity and extra residues attached to the N terminus of such proteins would disturb the correct conformation of their overall structure, abolish their biological function, or even induce protein degradation. In PRC1-DD, the first residue M1 is crucial in maintaining overall structural integrity by stretching into the protein hydrophobic core, stabilizing the core by making extensive interactions with other core residues. M1 is crucial in inducing the structural differences between the solution and crystal structures, not only in the N terminus but also in helix H2. The core-stabilizing M1 residue interacts with residues L50, M53 and E57 on H2; the key differences in H2 between the crystal and solution structures were found to be precisely these residues, with L50, M53 and E57 being core constituents according to solution structure and L51, M54 and E58 being core-composing residues of the crystal structure. Therefore, there is a strong possibility that the structural discrepancies between the solution and crystal structures are the result of the hydrophobic core placing of M1.

Mutating M1 adversely affected both the structure and the stability of the overall protein (Figure 6 and Figure 7). The construct used for crystallization had an N-terminal tag attached to the N terminus of PRC1, while the spatial restraint generated from the tag prevented M1 from stretching into the protein core, thus altering the correct core packing and bringing forth the observed differences between the solution structure and the corresponding segment of the crystal structure (Figure 5). Adding N-terminal extensions to PRC1-DD (mutants PRC1-DD-N+4 and PRC1-DD-N+His) produced very low protein expression profiles (Figure S7) and significantly reduced protein stability (Figure 8). It has been widely accepted that there is a positive correlation between protein stability and expression levels. The extremely low protein expression levels of PRC1-DD-N+4 and PRC1-DD-N+His might be attributable to their low protein stability, further supporting the crucial role that N-terminal residues play in maintaining a stable core. N-terminal extension also disrupted the overall structure, even producing an aggregatory (Figure 6) effect on the protein. Protein aggregation might be the result of improper folding, implying that residue M1 may also play a part in the folding of the protein.

In addition, in order to assess the effect of N-terminal extension on full-length PRC1, we also incorporated sequence ‘GAAA’ and His-tag into the N terminus of PRC1 1-486, generating mutants PRC1 1-486-N+4 and PRC1 1-486-N+His. They both showed sharp decreases in thermal stability compared with wild-type PRC1 1-486 (Figure 8 and Table 3), indicating that extra sequences attached to the N terminus of PRC1 indeed greatly affected the overall protein stability and possibly the whole protein structure.

The structural significance of the special N-terminal conformation of PRC1 could be unraveled; the core-placing M1 residue attributes a more compact and globular shape to the protein, incorporating more residues into the hydrophobic surface and creating a larger and stronger hydrophobic core, more efficiently stabilizing the protein.

## 5. Conclusions

In this study, we clarify the previous controversies regarding the multimerizational state of PRC1 by confirming that PRC1-DD exists as stable dimers under solution conditions. We also report the first solution structure of the dimerization domain of PRC1, PRC1-DD, which is also the first solution structure in the MAP family. Comparing the solution structure and the corresponding segment in the crystal structures of PRC1, the two are similar in terms of overall fold, but conformational differences in the form of altered backbone or hydrophobic core compositions are evident. The most significant differences between the two structures are the N-terminal residues, loop area and residues E49–E58 on helix H2. Among these, the conformation of N-terminal residue M1 is a unique discovery in our study, with M1 being buried in the protein hydrophobic core and having extensive interactions with other core residues; furthermore, M1 is the key to the differences between the solution and crystal structures, because the crystal structure has an extra N-terminal extension that might interfere with the conformation of M1 and, thus, the whole structure. We determined the structural significance of M1 by rationally designing a series of mutants, and we found that the core-stabilizing conformation of M1 shown in the solution structure is crucial to the structural integrity and stability of both PRC1-DD and full-length PRC1. Moreover, the structural differences in H2 between the solution and crystal structures might also be the direct consequence of M1 conformation.

This study suggests that the solution structure differs from the corresponding segment in the 3.2 Å X-ray crystal structure of PRC1 and more accurately describes the conformation that PRC1-DD adopts under solution conditions (Figure 5). An important lesson to be learned from this work is that the N terminus might be indispensable to the correct core packing and functional significance of the whole protein.

## Figures and Tables

**Table 1:** NMR structure and refinement statistics of PRC1-DD.

NMR Restraints	
Distance Restraints (NOE)	
Intraresidue (i = j)	674
Medium range (|i − j| < 5)	880
Long range (|i − j| > 5)	646
Ambiguous	1517
Strictly intermolecular	120
Total	3837
**Dihedral restraints (TALOS)**	
Φ	128
Ψ	128
Total	256
**Structure Statistics**	
**Ensemble RMSD**	
Backbone heavy atoms (Å)	0.37
All heavy atoms	0.55
All atoms (Å)	0.98
**NOE Violations**	
0.3 Å	0
0.2 Å	5
**AMBER Energy**	
Mean AMBER energy	−5060
**Ramachandran** ^1^	
Most favorable (%)	92.4
Additionally (%)	7.1
Generously Allowed (%)	0.5
Disallowed (%)	0

^1^ Calculated using PROCHECK.

**Table 2:** Tm values of PRC1-DD and its mutants.

Construct	Tm (1)	Tm (2)	Tm (3)	Tm (4)	Average Tm	Stdev
1_64 WT	49.42	49.23	49.48	49.62	49.44	0.16
1_64 N+4	41.20	41.60	40.80	41.90	41.38	0.48
1_64 Δ1R2M	36.36	37.14	37.81	38.13	37.36	0.78
1_64 N+His	<25	<25	<25	<25	<25	0.00
1_64 R2E	41.20	41.60	41.20	41.60	41.40	0.23

**Table 3:** Tm values of PRC1 1-486 and its mutants.

Construct	Tm (1)	Tm (2)	Tm (3)	Tm (4)	Average Tm	Stdev
1_486 WT	46.29	46.51	46.75	47.03	46.65	0.32
1_486 Δ1R2M	45.71	44.95	45.07	44.91	45.16	0.37
1_486 R2E	44.96	45.46	45.46	45.69	45.39	0.31
1_486 N+4	46.04	45.82	46.12	45.92	45.98	0.13
1_486 N-His	45.63	45.96	45.42	45.26	45.57	0.30

## Data Availability

The structure data used in this article can be found in the Protein Data Bank, PDB ID 7VBG; the NMR assignment data are available in the Biological Magnetic Resonance Data Bank (BMRB), entry number 51070.

## Supplementary Materials

The following are available at https://www.mdpi.com/article/10.3390/cimb1010000/s1, Figure S1: PRC1-DD in High Performance Liquid Chromatography. Figure S2: Assessing the molecular weight of PRC1-DD. Figure S3: Two-dimensional ${}^{1}H{-}^{15}N$ HSQC spectrum of PRC1-DD under different conditions. Figure S4: The RMSD values of PRC-DD solution and crystal structures under different alignment conditions. Figure S5: Slices from Three-dimensional ${}^{1}H{-}^{13}C$ NOESY-HSQC spectrum of PRC1-DD that show characteristic cross-peaks from residues that possess distinctive conformations in the solution and crystal structures. Figure S6: Structural variation of PRC1-DD mutants mapped to structure. Figure S7: SDS-PAGE gel showing the expression levels of PRC1-DD WT, mutant PRC1-DD-N+4 and PRC1-DD-His-N. Figure S8: Crystal structure B-factor of each residue. Table S1: SASA values of solution and crystal structures. Table S2: Distances of atoms from residues that have variations in conformation between solution and crystal structure. Table S3: Table showing the primer sequences of wild-type and mutants.

## Abbreviations

The following abbreviations are used in this manuscript:

MAP	Microtubule-associated protein
PRC1-DD	65 N-terminal residues of PRC1
SASA	Solvent-accessible surface area
RMSD	Root-mean-square deviation
FTSA	Fluorescence thermal shift assay
EM	Electron microscopy
Tm	Mid-denaturation temperature
HDX	Hydrogen–deuterium exchange experiment
SI	Supplementary Information
