# Supplementary material for: N-Terminus-Mediated Solution Structure of Dimerization Domain of PRC1
Source: Curr Issues Mol Biol. 2022 Apr 10;44(4):1626–45. doi: 10.3390/cimb44040111 (PMC9164050; doi:10.3390/cimb44040111)
Supplement: Supplementary file 1 [file cimb-44-00111-s001.zip › cimb-1641938-supplementary.pdf]

Supplementary Information of the Article “N-terminus Mediated Solution Structure of PRC1”

Fei Tan [1] and Jin Xu [1,*]

[1] Peking University

* Correspondence: tanfei777@pku.edu.cn; jxu@pku.edu.cn;

S1. Multimerizational State of PRC1-DD

We determined the molecular weight of PRC1-DD through Chemical crosslinking, High Performance Liquid chromatography (HPLC), and Static light scattering, the result of all of these experiments indicate that PRC1-DD exist as stable dimers in solution (Figure S1).

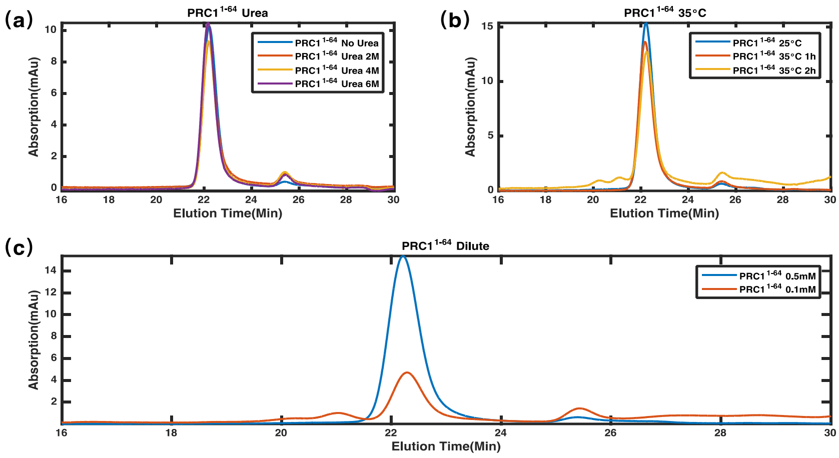

Figure S1. PRC1-DD in High Performance Liquid Chromatography (HPLC) under different conditions. **(a)** PRC1-DD in different concentration of urea. **(b)** PRC1-DD under different temperatures. **(c)** PRC1-DD at different concentrations.

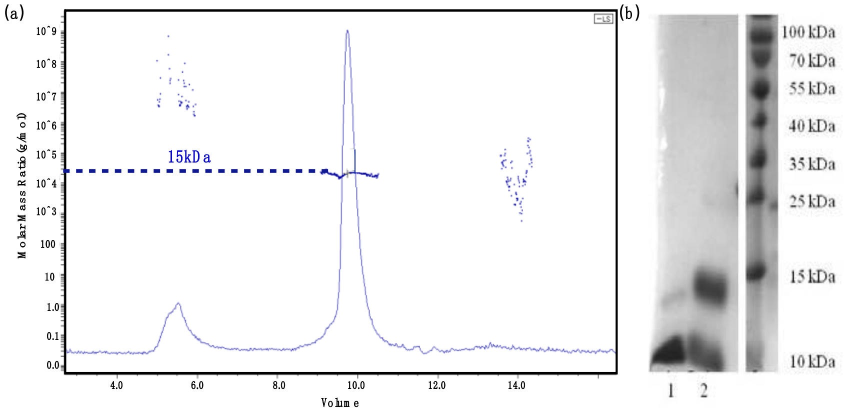

Figure S2. Assessing the molecular weight of PRC1-DD. **(a)** Static Light Scattering spectrum of PRC1-DD. **(b)** Chemical crosslinking result of PRC1-DD, in which lane 1 has no crosslinker added, lane 2 is the state of PRC1-DD with crosslinker.

S2. Stability of PRC1-DD

We recorded 2D ^{1}H-^{15}N-HSQC spectrum of PRC1-DD in solution under different experimental conditions. The 2D ^{1}H-^{15}N-HSQC spectrum of a protein is sensitive to changes in the protein structure, therefore, 2D ^{1}H-^{15}N-HSQC spectrum can be used to determine the solution conditions of PRC-DD suitable for NMR experiments. By recording the 2D ^{1}H-^{15}N-HSQC spectrum of PRC1-DD at different timepoints, we found that PRC1-DD can stay stable for up to one week at 25 ° C, as shown in Figure S3. Comparing the 2D ^{1}H-^{15}N-HSQC spectrum of PRC1-DD at 25°C and 35°C, we did not observe additional spectral peaks or large changes in chemical shifts, therefore, we can conclude that PRC1-DD is temperature insensitive (Figure S3 (b)).

To evaluate the stability of PRC1-DD, we measured the changes in the structure of PRC1-DD with or without urea. The 2D ^{1}H-^{15}N-HSQC spectra of PRC1-DD under 6M urea remain relatively unchanged, only when the urea concentration is as high as 8M (Figure S3 (f)) can we observe some irregular signals in the 2D ^{1}H-^{15}N-HSQC spectra of PRC1-DD.

Although PRC1-DD is an independent domain in terms of structure and function, but manual truncation might bring a certain degree of self-polymerization inclination, therfore, we also mesasured the self-polymerization potential of PRC1-DD. Generally, if a protein tend to self-polymerize, when the protein concentration is low, the peak intensities in its 2D ^{1}H-^{15}N-HSQC spectrum will be relatively uniform, but when the protein concentration is high, the relative intensities of spectral peaks will change[1]. We superimposed and compared PRC1-DD in different concentrations (0.1 mM and 0.5 mM), and we found that no concentration related change in peak intensities of amides could be observed (Fig. 3.14 (c), (d)), so we believe that no concentration-dependent oligomerization of PRC1-DD occurred.

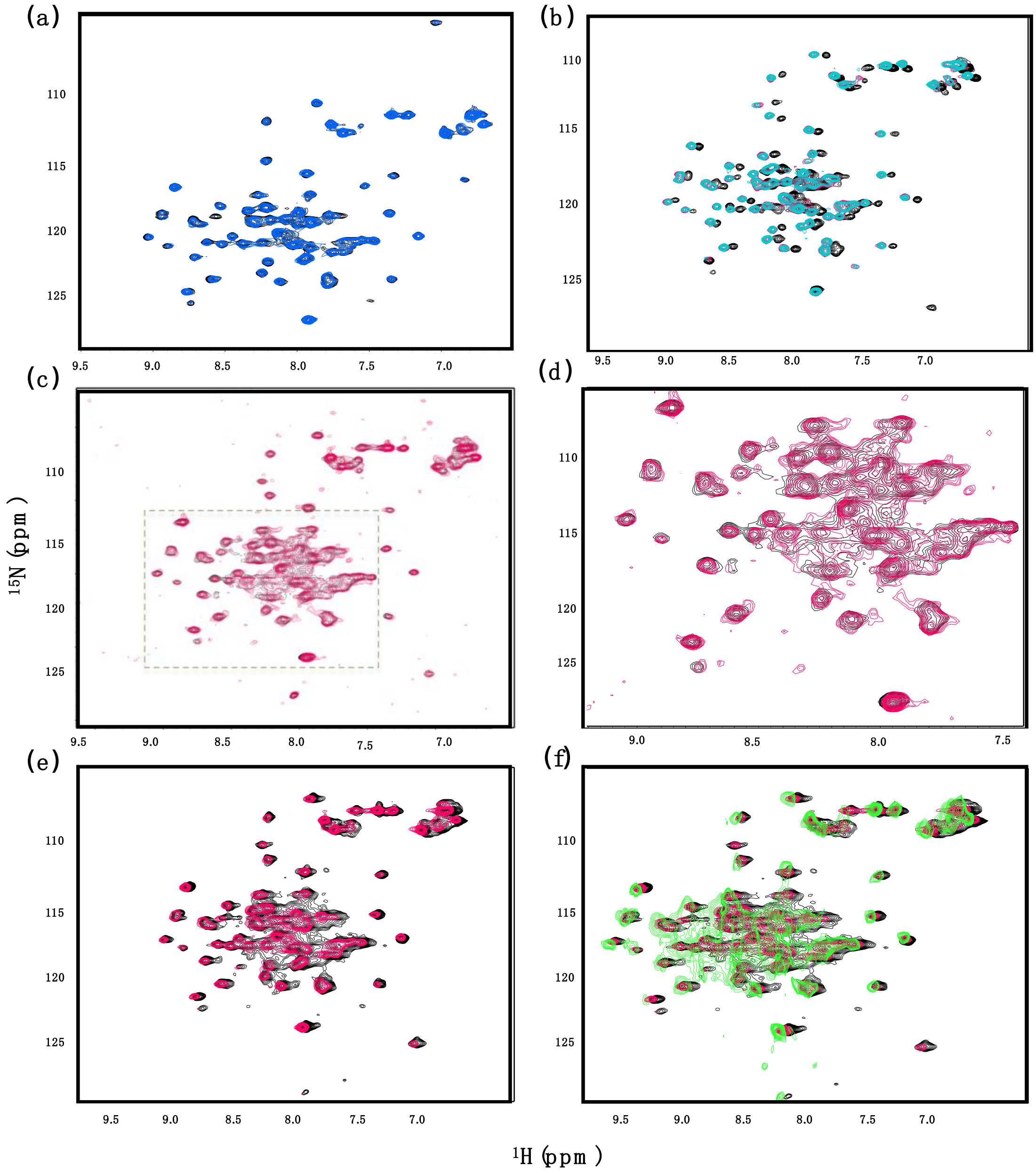

Figure S3. 2D ^{1}H-^{15}N-HSQC spectra of PRC1-DD under different conditions. **(a)** PRC1-DD (black) compared with itself after 7 days (blue). **(b)** PRC1-DD under 20°C (black), 25°C (blue) and 35°C (green). **(c)** PRC1-DD at 0.5mM (black) compared with itself at 0.1mM (red). **(d)** enlargement of the box in (c). **(e)** PRC1-DD (black) and with 6M urea. **(f)** PRC1-DD (black) and with 8M urea.

S3. Different Methods of Alignment Between the the Solution and Crystal Structures

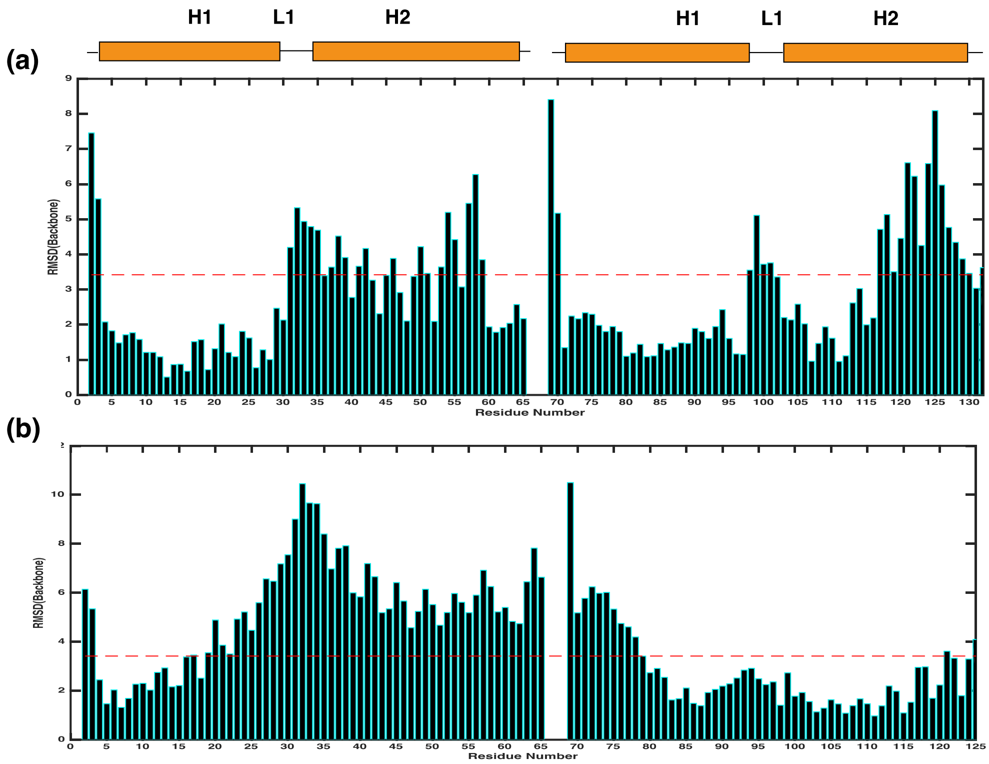

Figure S4. The RMSD values of PRC-DD solution and crystal structures under different alignment conditions. **(a)** aligning H1s of the two monomeric units. **(b)** aligning a single monomeric unit in the homodimer. Residue number 1-67 denote the first monomeric unit, while 67-134 denote the second monomeric unit.

S4. NOESY Cross-peaks Demonstrating the Different Conformations of the Solution and Crystal Structures

Proton-proton distance information is the basis for calculating the solution structure. We obtained proton-proton distance information of PRC1-DD mainly from its 3D NOESY spectrum., therefore, the differences between the solution and crystal structure are mainly due to the characteristic distance constraints obtained from the 3D NOESY spectrum of PRC1-DD.

In Fig S5, we present slices of 3D ^{1}H-^{13}C NOESY-HSQC spectrum of PRC1-DD. We have selected slices of the spectrum with the criterion: first, only those residues whose backbone or side chain conformations possess significant differences between the solution and the crystal structures. Second, the chemical assignments of these cross-peaks possess few or no overlap. The existence of a spectral peak in 3D ^{1}H-^{13}C NOESY-HSQC spectrum implies that the spatial distance between the two atoms in the solution structure is small (< 5 Å), but according to the crystal structure, the distance between the two designated atoms far exceeds the upper limit (5 Å) of distances that can be measured by the NOESY experiment. Therefore, the existence of these cross-peaks alone is sufficient to prove that there are significant differences between the solution structure and the crystal structure. Table S2 lists the

interatomic distances measured from the solution structure and the crystal structure of PRC1-DD respectively. The residue numbers of the first subunit is 1-64, and the residue numbers of the second subunit is 68-131.

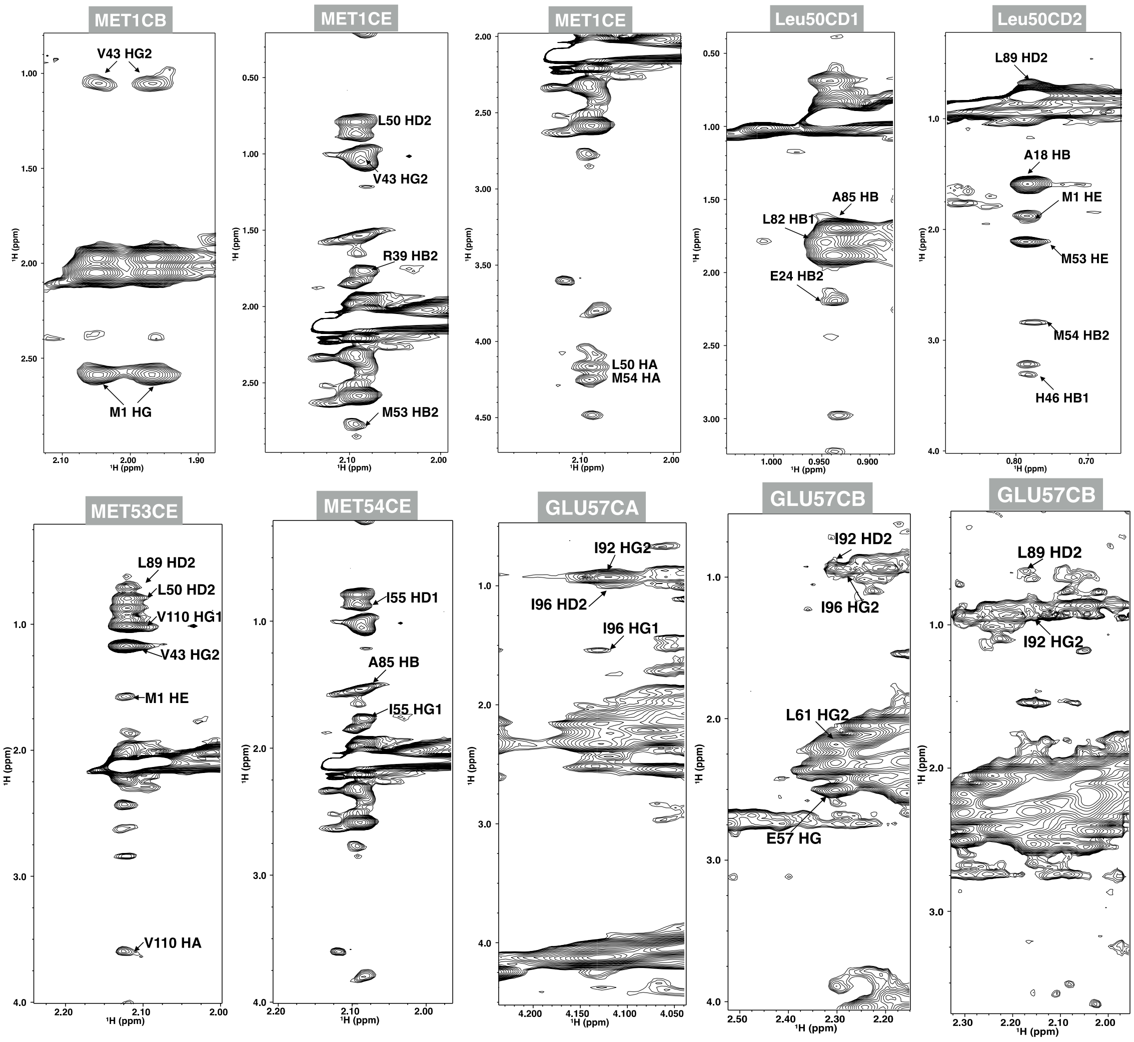

Figure S5. Slices from 3D ^{1}H-^{13}C NOESY-HSQC spectrum of PRC1-DD that show characteristic cross-peaks from residues that possess distinctive conformations in the solution and crystal structures.

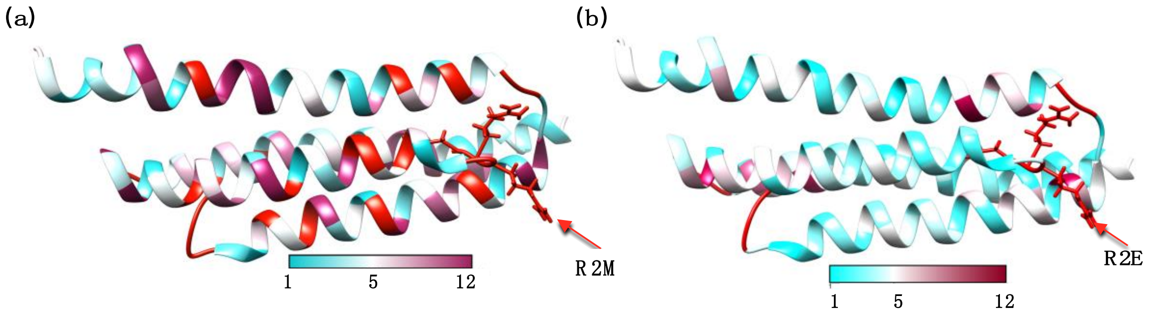

Figure S6. Structural variation of PRC1-DD mutants mapped onto structure, residue R2 is represented in ball-and-stick representation. **(a)** For mutant Δ1R2M, residues with great chemical shift changes are mapped to PRC1-DD structure. **(b)** For mutant R2E, areas with great chemical shift changes are mapped to PRC1-DD structure. Red denote areas with chemical shift change of 8ppm or more, white denotes chemical shift change of around 5 ppm, blue shows no chemical shift change.

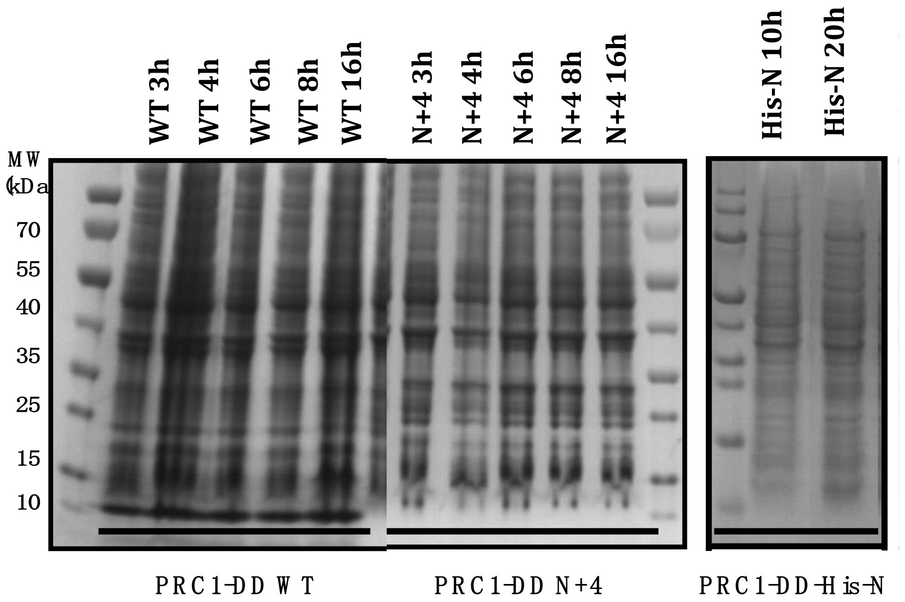

Figure S7. SDS-PAGE gel showing the expression levels of PRC1-DD WT, mutant PRC1-DD-N+4 and PRC1-DD-His-N (with tag "GAAA" and his-tag incorporated into the N terminal of PRC1-DD respectively), each lane shows the eluted protein profile with induction time labeled on top of each lane. The expression system are *E.Coli /Rosseta* (DE3). The molecular weight of PRC1-DD is around 15kD.

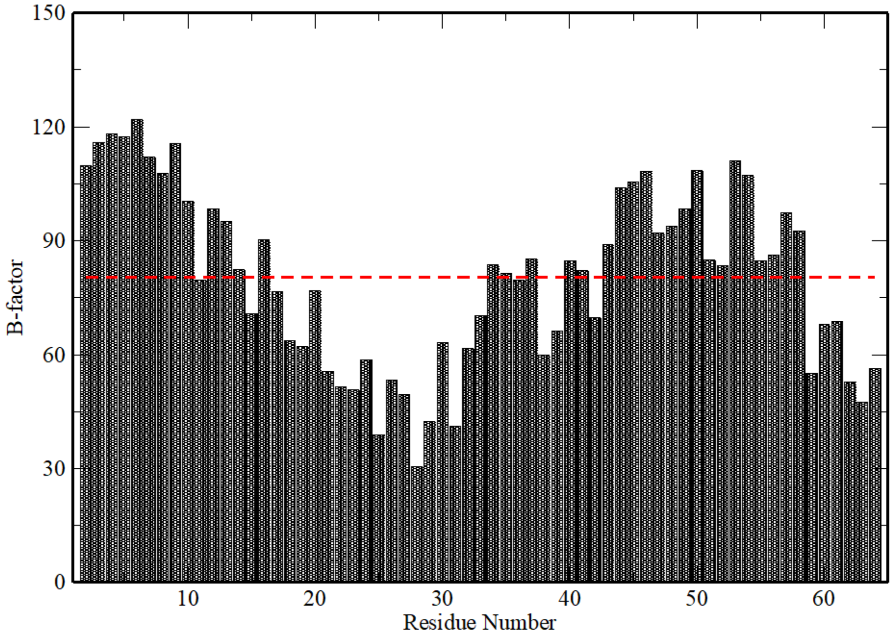

Figure S8. Crystal structure B-factor of each residue, values shown here were taken from PDB ID:4L6Y.

Table S1. SASA values of solution and crystal structures

Sequence	Residue	Solution Structure	Crystal Structure
**1**	MET	N/A	50.20%
**2**	ARG+	60.80%	52.30%
**3**	ARG+	48.50%	67.90%
**4**	SER	3.40%	23.60%
**5**	GLU	19.30%	27.90%
**6**	VAL	29.60%	45.40%
**7**	LEU	10.10%	52.00%
**8**	ALA	7.20%	7.80%
**9**	GLU	33.90%	33.40%
**10**	GLU	26.50%	34.00%
**11**	SER	0.20%	27.60%
**12**	ILE	11.40%	32.70%
**13**	VAL	36.40%	28.70%
**14**	CYS	12.70%	18.00%
**15**	LEU	0.00%	21.10%
**16**	GLN	19.60%	39.90%
**17**	LYS+	34.50%	42.50%

**18**	ALA	5.50%	19.00%
**19**	LEU	2.80%	31.70%
**20**	ASN	17.30%	24.80%
**21**	HIS+	11.60%	33.70%
**22**	LEU	0.40%	25.80%
**23**	ARG+	22.00%	29.70%
**24**	GLU	24.20%	29.50%
**25**	ILE	0.00%	16.40%
**26**	TRP	2.60%	23.20%
**27**	GLU	33.30%	37.30%
**28**	LEU	37.70%	50.10%
**29**	ILE	16.70%	32.00%
**30**	GLY	45.50%	29.90%
**31**	ILE	9.60%	22.50%
**32**	PRO	28.40%	35.20%
**33**	GLU	21.70%	32.00%
**34**	ASP	28.10%	29.70%
**35**	GLN	34.20%	23.50%
**36**	ARG+	6.30%	9.50%
**37**	LEU	6.00%	40.90%
**38**	GLN	35.30%	35.90%
**39**	ARG+	17.30%	30.60%
**40**	THR	0.80%	29.40%
**41**	GLU	22.70%	23.20%
**42**	VAL	29.90%	27.80%
**43**	VAL	0.40%	18.30%
**44**	LYS+	13.70%	32.30%
**45**	LYS+	42.40%	40.40%
**46**	HIS+	21.90%	36.20%
**47**	ILE	0.30%	27.10%
**48**	LYS+	36.70%	32.10%
**49**	GLU	27.10%	38.90%
**50**	LEU	11.80%	20.30%
**51**	LEU	1.10%	21.30%
**52**	ASP	26.20%	27.60%
**53**	MET	38.20%	30.50%
**54**	MET	10.20%	27.50%

**55**	ILE	8.80%	35.20%
**56**	ALA	26.00%	18.50%
**57**	GLU	35.10%	28.10%
**58**	GLU	6.10%	36.20%
**59**	GLU	17.20%	38.00%
**60**	SER	19.70%	25.30%
**61**	LEU	27.60%	30.90%
**62**	LYS+	20.00%	33.40%
**63**	GLU	42.60%	22.00%
**64**	ARG+	48.70%	45.50%
**65**	LEU	79.10%	52.40%

Table S2. Distances of atoms from residues that have variations in conformation between solution and crystal structure

**Atom 1**	**Atom2**	**Solution Structure（Å）**	**Crystal Structure（Å）**
1 MET HB	110 VAL HG	4.8	>8
1 MET HE	50 LEU HD	2.5	>8
1 MET HE	50 LEU HA	4.5	>8
1 MET HE	110 VAL HG2	2.3	>8
1 MET HE	54 MET HA	4.9	>8
1 MET HE	53 MET HB2	4.2	>8
1 MET HE	106 ARG HB	4.3	>8
18 ALA HB	117 LEU HD1	2.6	8.5
19 LEU HD1	40 THR HG	4.9	6.3
50 LEU HD1	53 MET HE	3.3	8.6
50 LEU HD1	54 MET HE	3.4	6.5
50 LEU HD1	51 LEU HD2	4.1	5.7
50 LEU HD1	85 ALA HB	2.5	8.3
50 LEU HD2	82 LEU HD1	2.9	7.4
50 LEU HD2	89 LEU HD1	2.5	9.3
50 LEU HD2	53 MET HE	2.5	6.2
50 LEU HD2	54 MET HE	4.1	5.8
51 LEU HD1	53 MET HE	3.8	9.4

51 LEU HD1	54 MET HE	2.7	4.9
53 MET HE	89 LEU HD1	2.4	9.3
53 MET HE	89 LEU HD2	2.6	8.5
53 MET HE	110 VAL HG2	4.9	11
53 MET HE	107 THR HN	4.0	12
54 MET HE	55 ILE HG	4.4	6.8
54 MET HE	55 ILE HD	2.4	7.7
54 MET HE	85 ALA HB	2.9	7.5
57 GLU HA	92 ILE HG2	3.9	8.4
57 GLU HA	96 ILE HD1	4.0	7.4
57 GLU HB	89 LEU HD2	3.0	6.0
57 GLU HB	92 ILE HG2	3.9	7.5
57 GLU HB	96 ILE HD1 /HG2	3.5	8.2/9.7
57 GLU HB	103 ARG NH1	3.0	10.5
57 GLU HB	106 ARG NH1	4.3	6.0

Table S3. Table showing the primer sequences of wild-type and mutants (F is for forward primer, R is for reverse primer, restriction enzyme sites were indicated in the name of each primer)

Construct	Primer
**F_Nde1**	GGCATTCCATATGATGAGGAGAAGTGAGGTGCTG
**486_R_Xhol**	CCGCTCGAGACGTGCTTTGCCCGGTGTATT
**DD_R_Xhol**	GAAAGACTCCATCATCACCATCACCATUAGCCTCGAGG
**R2E/F**	AGAAGGAGATATACATATGGAGAGAAGTGAGGTGCTG
**R2E/R**	CAGCACCTCACTTCTCTCCATATGTATATCTCCTTCT
**R2M/F**	AGAAGGAGATATACATAGGATGAGAAGTGAGGTGCTGG
**R2M/R**	CCAGCACCTCACTTCTCATCCTATGTATATCTCCTTCT

**N+4/F**	AGAAGGAGATATACATATGGGCGCAGCAGCAATGAGGAGAAGTGAGGTG
**N+4/R**	CACCTCACTTCTCCTCATTGCTGCTGCGCCCATATGTATATCTCCTTCT
